# The Effect of Abutment Angulation and Crown Material Compositions on Stress Distribution in 3-Unit Fixed Implant-Supported Prostheses: A Finite Element Analysis

**DOI:** 10.1155/2022/4451810

**Published:** 2022-08-27

**Authors:** Ramin Mosharraf, Mahsa Abbasi, Pirooz Givehchian

**Affiliations:** ^1^Dental Material Research Center and Department of Prosthodontics, School of Dentistry, Isfahan University of Medical Sciences, Isfahan, Iran; ^2^Department of Prosthodontics, School of Dentistry, Shahrekord University of Medical Sciences, Shahrekord, Iran; ^3^Dental Implants Research Center, Dental Research Institute, School of Dentistry, Isfahan University of Medical Sciences, Isfahan, Iran

## Abstract

**Objective:**

The aim of this study was to evaluate influence of abutment angulation and restoration material compositions on the stress pattern in dental implants and their surrounding bone.

**Materials and Methods:**

In this finite element study, the six different solid 3D models of “mandibular 3-unit fixed implant-supported prostheses” were analyzed. In all of these models, a straight abutment was used for anterior implants at the second premolar site, and in order to posterior implant at the second molar site, abutments with three different angles (straight, 15, and 20°) were used. Also, two different restoration material compositions (porcelain fused to base metal (PFBM) and porcelain fused to noble metal (PFNM)) were considered for fixed implant supported restorations. A 450 N static force was exerted in a straight manner along the longitudinal axis of the anterior implant in a tripod, and the stress distribution was measured based on the restoration materials and abutment angulations of the models in the 3 sites of cortical, cancellous bone, and fixtures. The simulation was performed with ABAQUS 6.13 Software.

**Results:**

In all models, stress values in surrounding cortical bone were more than in spongy bone. Maximum stress levels in an anterior abutment-implant complex were seen in models with angled implants. In models with parallel implants, the stress level of a molar straight abutment-implant complex was less than that of premolar straight ones. In an angled posterior abutment-implant complex, less stress level was detected compared to straight ones. In all PFNB models, stress values were slightly more and distributed in a wider area of premolar straight abutments.

**Conclusion:**

Increasing an abutment angle, increases stress in surrounding bone and straight implant-abutment combination. It seems that the crown material composition affects stress distribution of the implant-abutment combination but does not affect stress distribution of surrounding bone.

## 1. Introduction

A high success rate of dental implants has always been an attraction for clinicians, especially for posterior teeth which can face fracture the most [1]. Dental implants are mostly intended to be installed parallel to other teeth and also each other, but in some clinical conditions, installing implants in such a way is impossible, so angled abutments are a common solution [2]. Use of angled abutments varies stress distribution on both implants and its surrounding bone [3, 4]. Furthermore, restoration material compositions (coping and veneering porcelain) could affect stress distribution [5]. In order to determine lifetime and clinical reliability of implant supported fixed prosthesis, it is essential to have accurate knowledge of stress distribution on implants and its surrounding bone [6]. The finite element method (FEM) along with computer-aided design (CAD) is an effective method to estimate the stress distribution by different loadings, prosthesis geometries, and restoration material compositions.

Some previous studies reported that by increasing abutment angulation, both stress and strain levels in the implant and the rate of bone stress will increase [7, 8]. Yang et al. [9] stated that with the increase in the abutment angulation from 15° to 20°, the bone and bolt stress increase significantly. Martini et al. [10] in their study showed the maximum cortical bone stress obtained in the angulated abutment group in comparison to the straight abutment group. Tian et al. [11] stated that angulated abutments may decrease the stress rate in surrounding bone.

Behnaz et al. [12] reported that if force loading was parallel to implant angulation, bone and implant stress may decrease.

About the effect of restoration materials on stress distribution, various results were found. Meric et al. [5] reported that design and material composition of prosthesis in three-unit implant-supported prostheses affect force distribution. Sevimay et al. [13] stated that increasing rigidity of material for the implant superstructure does not affect the stress level and distribution of bone surrounding implants but it could be different for abutment and crown structure patterns. They showed that the porcelain fused to base metal causes higher stress than the porcelain fused to noble metal crown. Also, Bacchi et al. study [14] showed that the noble metal in comparison to the base metal makes a lower amount of stress in the prosthesis framework. The results of Lambodaran et al. study [15] showed that however different framework material result in different amount of stress values but none of them did not create amount of stress that would be harmful for implant and it's surronding bone.

According to the different results of previous studies about the level and distribution of stress in implants and bone in different abutment angulation and prosthesis materials, more studies needed to be performed in this context. The aim of the present study was to assess the effect of the abutment angulation and different materials used for implant-supported prostheses on stress distribution in the implant and surrounding tissues by finite element analysis (FEA). The null hypothesis of the study was that the abutment angulation and different materials used for implant-supported prostheses could not affect the stress distribution.

## 2. Materials and Methods

In this experimental study, to define geometry of analysis, 3D scanning technology (Solutionix Rexcan III, Seongbuk-gu, Seoul, South Korea) was used.

In a mandibular-prefabricated edentulous model, in the region of the right second premolar and the second molar, two-piece bone level implants (Biohorizons Internal, Implant system Inc., Birmingham, Al, USA) (Ø 4.1 mm, 10 mm long) were inserted parallel to each other by a dental surveyor (Ney surveyor, Dentsply Intl, York, Pa).

The 3D geometry of this model, fixture, and straight, 15°, and 20° angled Biohorizon abutments (4.5 platform, collar 1 and 3) was scanned.

The abutments were placed on the model, and a Ni– Cr (Verabond-II, AalbaDent, Inc., CA, USA) 3 unit coping with a thickness of 0.5 mm was cast and scanned. Veneering porcelain (VITA Zahnfabrik, Bad Sackingen, Germany) with a thickness of 1 mm was applied on it, and therefore, a three-unit fixed partial denture (FPD) was developed and rescanned. The same process was performed to fabricate another FPD by using a noble metal.

The models were assembled by the Catia software (V5R21, BM, Kingstone, NY, USA). The implant bone and crown-abutment interface were assumed to be completely bonded.

**Figure 1:**
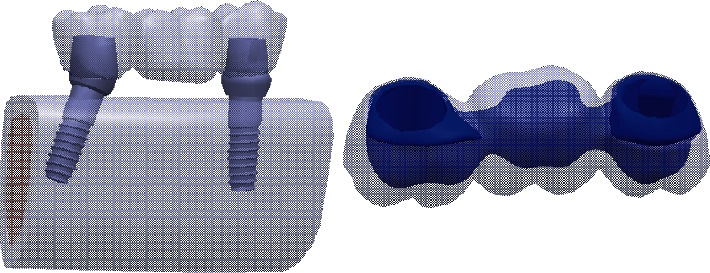
3D model of implant angulation and three-unit FPD.

Three models were designed by computerized modeling. The anterior implant was inserted in a straight manner, and a straight abutment was used for it. Implant in second molar site (Figure 1):

- Implant in second molar site in model 1 was inserted straightly and a straight abutment was inserted.
- Implant in second molar site in model 2 was inserted with a 15° mesial angulation and a 15° angled abutment was placed on the it.
- Implant in second molar site in model 3 was inserted with a 20° mesial angulation and a 20° angled abutment was placed on the it. Finally, six virtual models were constructed by using 2 types of 3-unit implant-supported restoration material as mentioned in (Figure 1).

In all of the six models, thickness of cortical bone was 2 mm, and in the center of that cancellous bone was assembled.

The digital 3D scanner created point clouds. These point clouds were then converted to surfaces using Geomagic7 (3D systems USA) software. After editing, the created surfaces were converted to 3D solid parts, and using CATIA v5 assembly design, components were primarily assembled and a 3D solid model was developed. This model was then imported to the ABAQUS v.6.13 finite element package as an assembly file.

All materials used in this study were considered to be isotropic.  Table 1 shows material properties used in this study.

**Figure 2:**
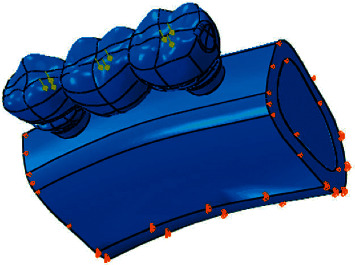
Tripod loading.

Components were tied to each other to create a uniform component. A feature of the FEA is that the reference plane for deformation must be defined to remove possible rigid body motion; therefore, the mandibular lower border is considered as a boundary condition. To increase similarity of the developed model to the real model, the lower part of the model (bone) was completely fixed, and on the flat sides of the model, all displacements perpendicular to these flat surfaces were fixed; between all components with no displacement against each other like implant and bone, the “tie” constraint was applied as a boundary condition. Then, a 450 N static force was applied in a straight manner along the longitudinal axis of the anterior implant on three points of the fixed partial denture, including the central fossa in the second molar, the central fossa in the first molar, and the distal fossa in the second premolar, in a tripod manner (on three points, 150 N on each point) (Figure 2).

Six different models (different in material composition and abutment installation geometry) were created and meshed using the type C3D4R mesh. The geometry was meshed by tetrahedral elements with 4 nodes.  Table 2 shows model meshing specifications.

As the number of elements increases, the result becomes more accurate. In mesh processing of the present study, the number of elements was increased until the answers in each model converged to one number. After meshing, the developed model was solved and von Mises stresses were considered as the output of finite element analysis to compare different models.

Stress analysis was performed using the FEA software ABAQUS v.6.13 (ABAQUS Inc., Providence, RI).

## 3. Results

### 3.1. Stress Distribution in Surrounding Bone in Model 1

**Figure 3:**
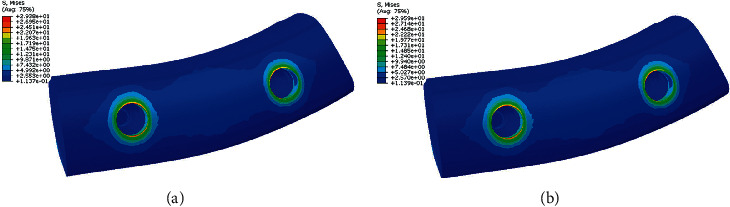
Stress distribution in bone in model 1. (a) PFBM. (b) PFNM.

Figure 3 shows stress distribution in cortical and spongy bone in two different materials of PFBM and PFNM. Stress values in cortical bone and spongy bone in both models were approximately similar. Also, in both models, stress values in cortical bone were more than in spongy bone. Stress values in both cortical and spongy bone surrounding premolars were less than around molars.

### 3.2. Stress Distribution in Abutments in Model 1

**Figure 4:**
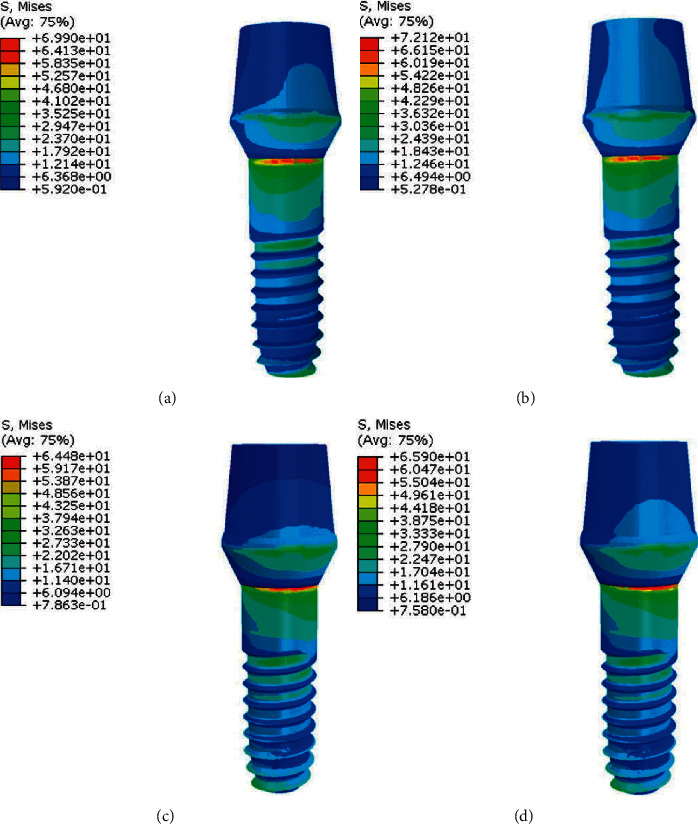
Stress distribution in abutments in model 1. (a), (c) PFBM; (b), (d) PFNM.

FEA results showed that in both material compositions, stress levels of the straight abutment installed in molar figures 4(c)and 4(d) are less than stress values in the straight abutment installed in premolar figures 4(a) and 4(b).  Figure 4 shows stress values and distribution in model 1.

### 3.3. Stress Distribution on Surrounding Bone in Model 2

**Figure 5:**
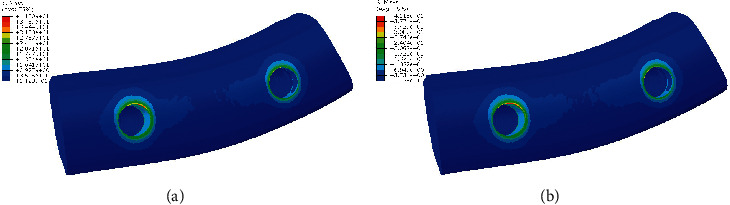
Stress distribution in bone in model 2. (a) PFBM. (b) PFNM.

Our findings showed that stress values in bone surrounding angulated implants in both material compositions were approximately equal. In both material compositions, stress values in cortical bone were more than stress values in spongy bone. Stress values in both cortical and spongy bone surrounding premolars were less than stress values around molars (Figure 5).

### 3.4. Stress Distribution in Abutments in Model 2

**Figure 6:**
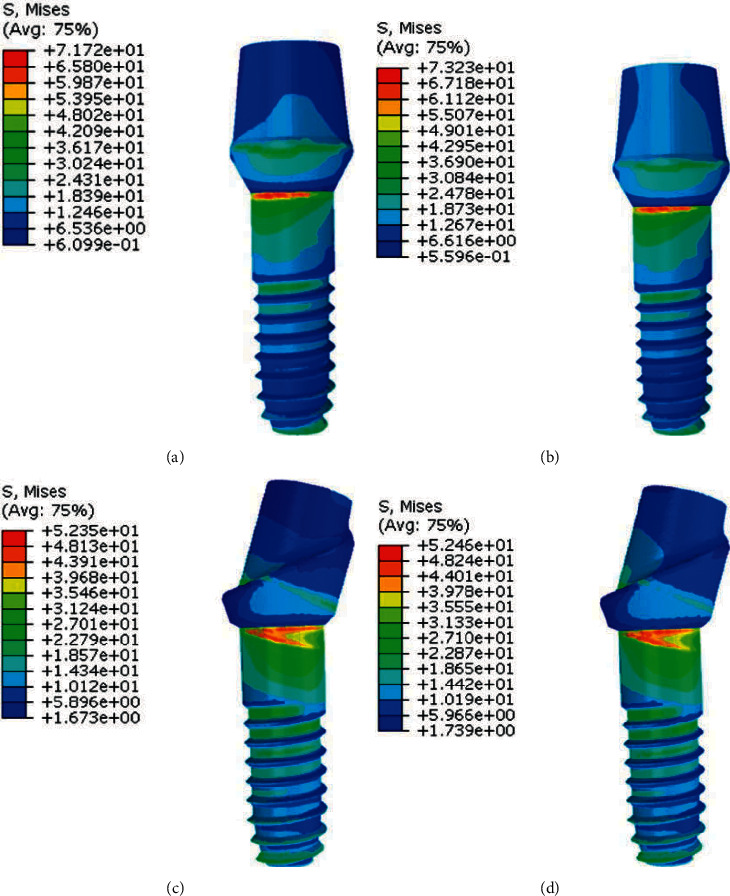
Stress distribution in abutments in model 2. (a), (c) PFBM; (b), (d) PFNM.

Figure 6 shows stress distribution in 15° angulated abutments for both PFBM and PFNM. It was shown that in both material in angled posterior abutments (Figures 6(c)and 6(d)), less stress values were applied compared to straight anterior abutments (Figures 6(a) and 6(b)).

### 3.5. Stress Distribution in Surrounding Bone in Model 3

**Figure 7:**
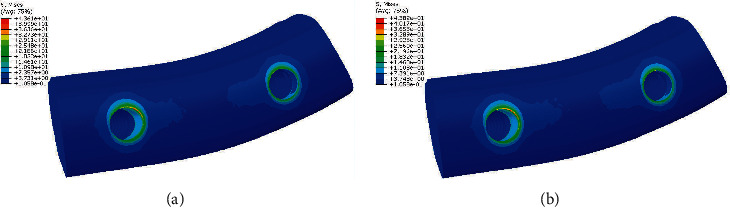
Stress distribution in bone in model 3. (a) PFBM. (b) PFNM.

Stress distribution in cortical and spongy bone surrounding anterior straight and posterior 20° angulated abutments in model 3 is shown in Figure 7. FEM analysis showed that in both material compositions, stress distribution was approximately the same. Also, in all sites, stress values in spongy bone were less than stress values in cortical bone. In addition, like previous models, stress values in both cortical and spongy bone surrounding premolars were less than around molars.

### 3.6. Stress Distribution in Abutments in Model 3

**Figure 8:**
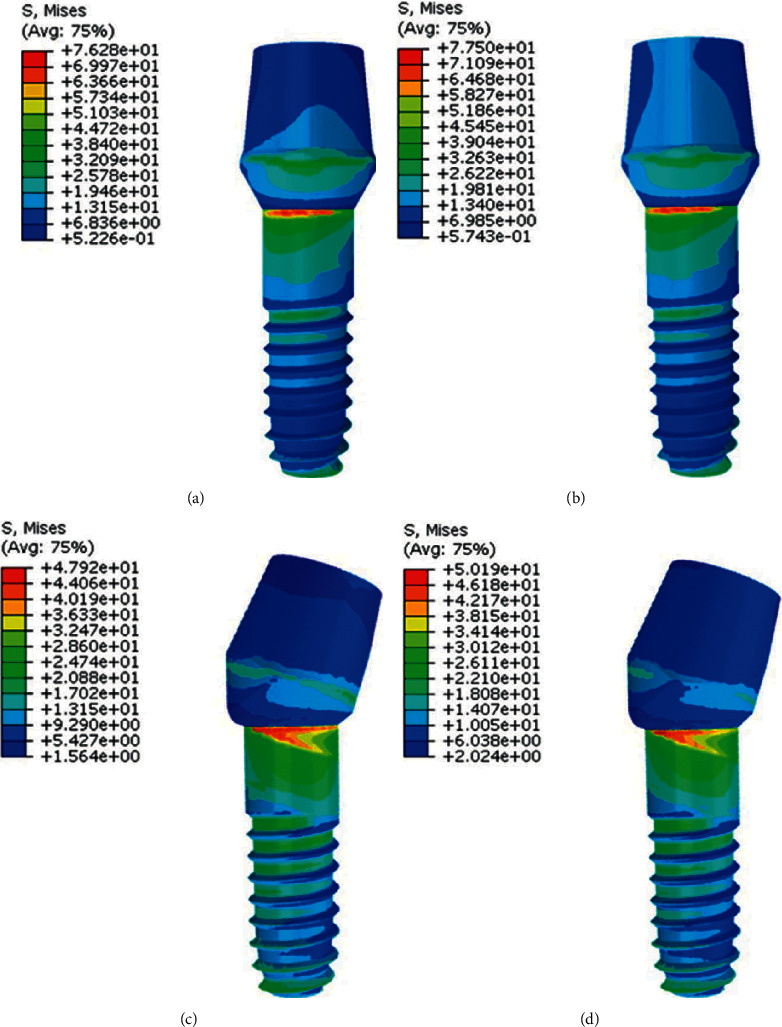
Stress distribution in abutments in model 3. (a), (c) PFBM; (b), (d) PFNM.

In Figure 8 stress values and stress distribution in anterior and posterior abutments for both material compositions are shown. Results showed that stress levels in posterior angulated abutments (Figures 8(c) and 8(d)) were less than in anterior straight abutments (Figures 8(a) and 8(b)).

## 4. Discussion

In the present study, the effect of the abutment angle and restoration material compositions on stress distribution in dental implants and surrounding bone was assessed. The null hypotheses were rejected. It seems that the crown material composition affects stress distribution of abutment-implant combinations but does not affect stress distribution of surrounding bone. However, implant angulation affected stress distributions in both of them.

The finite element method was used in this study because of its superiority in computer programming methods and digital imaging techniques [16]. The posterior mandibular region was simulated in this finite element analysis study. This region is a load-bearing region, and more stress can be anticipated in this region. [17].

Lambodaran et al. [15] showed in their study that metal ceramic restoration in comparison to gold porcelain creates a higher amount of stress. They also explained the differences in stress values among various materials can be attributed to the difference in the elastic modulus of different materials, with the rigid materials transferring minimal stress. Different results between the mentioned study and the present study could be related to the difference in material brands and the amount of applying force. Gomes et al. [18] reported similar results with the present study and stated that the use of different materials to fabricate a superstructure for a single implant-supported prosthesis does not affect the stress distribution in the supporting bone.

In all models, stress values in distal implants were less those in anterior straight implants in the premolar site. However, it was different for stress values of surrounding bone. As the implant angle was increased, the stress level in surrounding bone increased.

It seems that the direction of applying forces might play a role in stress distribution in surrounding bone. Wu et al. [19] stated that the magnitude of stress within peri-implant bone increased with an increase in the abutment angulation under axial loading; it was possible that the magnitude of stress within peri-implant bone increased or decreased with an increase in the abutment angulation under oblique loading. Also, Ebadian et al. [12] reported that angulation of implants can reduce stresses when the application of the load is in the same direction as the implant angulation.

Results show that although application of angled abutments reduces the stress value on angled implants itself, more stress is exerted on bone tissues surrounding implants and other FDP base implants. This difference in the stress value is referred to force direction, which was applied in the same direction of the longitudinal axis of the anterior straight abutment in the premolar site. Therefore, the greatest portion of force is exerted along the bone, and less stress is applied to the implant itself. Also, Ebadian et al. [12] reported that angulation of implants can reduce stresses when the application of the load is in the same direction as the implant angulation.

## 5. Conclusion

- It seems that the crown material composition affects stress distribution of implant-abutment combination but does not affect stress distribution of surrounding bone.
- Increasing the abutment angle increases stress in surrounding bone and straight implant-abutment combination.

## Figures and Tables

**Table 1:** Material properties.

	Material	Elastic modulus (MPa)	Poisson ratio
	Titanium	1.1 × 101^1^	0.35
	Cortical bone	1.37 × 10^10^	0.30
	Spongy bone	1.37 × 10^9^	0.30′
PFBM	Porcelain	8.28 × 10^10^	0.35
	Coping	2.06 × 10^11^	0.35
PFNM	Porcelain	8.28 × 10^10^	0.35
	Coping	8.95 × 10^11^	0.33

Porcelain fused to base metal (PFBM), porcelain fused to noble metal (PFNM).

**Table 2:** Model meshing specifications.

	Material composition	Abutment angle	Number of elements
1	PFBM	0	1814852
2	PFBM	15	1791452
3	PFBM	20	1771196
4	PFNM	0	1814852
5	PFNM	15	1791452
6	PFNM	20	1771196

## Data Availability

The supporting data are available at the library of Isfahan Dental School.
